# Choice of Treatment for Stage IA Non-small Cell Lung Cancer Patients Ineligible for Surgery: Ablation or Stereotactic Body Radiotherapy?

**DOI:** 10.7150/jca.39465

**Published:** 2020-01-14

**Authors:** Long Liang, Guoshu Li, Shuanshuan Xie, Guifeng Sun, Mengmei Zhang, Fenyong Sun, Aimei Peng

**Affiliations:** 1 Department of Respiratory Medicine, Shanghai Tenth People's Hospital, Tongji University, Shanghai 200072, China; 2 Department of Clinical Laboratory, Shanghai Tenth People's Hospital, Tongji University, Shanghai 200072, China

**Keywords:** Ablation, SBRT, non-small cell lung cancer, SEER, overall survival

## Abstract

**Purpose**: To compare the survival outcomes of ablation and stereotactic body radiotherapy (SBRT) in inoperable patients with stage IA non-small cell lung cancer (NSCLC).

**Patients and Methods**: Using the Surveillance, Epidemiology, and End Results (SEER) database, we identified 6,395 patients with stage IA NSCLC who had complete clinical information from 2004 to 2015. Kaplan-Meier analysis was performed to determine the propensity score based on the clinical characteristics of patients with stage IA NSCLC. Overall survival (OS) was compared between patients with stage IA NSCLC who were treated with ablation and SBRT after adjusting, stratifying, or matching.

**Results**: Kaplan-Meier analysis demonstrated no significant difference in survival curves (log-rank, *p*>0.05) between the ablation and SBRT groups. Compared with the SBRT group, the hazard ratio (HR) (95% confidence interval [CI]) of OS was 0.930 (0.817-1.058, *p*=0.269) in the ablation group on univariate analysis. On multivariate analysis, similar effects on OS (HR: 0.974, 95% CI: 0.858-1.105,* p*=0.680) were seen in patients with stage IA NSCLC in both the groups.

**Conclusions**: This study suggests that survival does not differ significantly between patients with stage IA NSCLC treated with ablation and SBRT. These results will be helpful for patients with stage IA NSCLC who are ineligible for surgery.

## Introduction

According to the American Cancer Center, there were about 234,030 newly diagnosed lung cancer patients in 2018, of which more than 80% had non-small cell lung cancer (NSCLC)^1^,^2^. Early-stage lung cancer patients account for 16% of newly diagnosed cases, diagnosed according to the criteria of the American Joint Commission for Cancer stage I or II disease^3^. Currently, surgery is the gold standard for treatment of early-stage NSCLC^4^. However, >20% of patients with early-stage NSCLC are ineligible for surgery because of various factors such as old age, severe impairment of lung function, or other comorbidities^5^. Therefore, viable alternatives, including ablation and SBRT, have been emerging to achieve reliable local control in such patients^6^,^7^. Three-year local control rates of up to 90% were observed on application of SBRT for early-stage lung cancer^6^,^8^. Ablation, including laser ablation, cryotherapy, electrocautery, and fulguration, is an image-guided technique^9^. Good local-regional control has been reported with ablation, compared to that with SBRT, for patients with inoperable NSCLC^10^,^11^. To date, no large population-based studies have been performed using a cancer database to compare clinical outcomes between ablation and SBRT cohorts. In addition, there have been no randomized studies or prospective trials for assessing the effectiveness of the two treatments. The purpose of this study was to compare survival rates between patients with stage IA NSCLC treated with ablation and SBRT using the Surveillance, Epidemiology, and End Results (SEER) database.

## Patients and Methods

### Data Source

The data used in this study were extracted from the SEER database. The SEER database is sponsored by the US National Cancer Institute and collects registry information, including that of patient survival, pathological type, disease stage, and treatment. The SEER database was established in 1973 and contains data of approximately 10% of the US population.

### Study Population

We limited the cohort to patients diagnosed with stage IA NSCLC (tumor size ≤3 cm) between 2004 and 2015. All included patients were inoperable and underwent SBRT or ablation (including laser ablation, cryotherapy, electrocautery, and fulguration). Complete patient information was available in the SEER database.

### Covariates

Baseline characteristics were based on 14 covariates, including age, sex, tumor size, race, differentiation grade, tumor location, histologic type, laterality, insurance status, marital status, year of diagnosis, geographic region, education level, and median household income.

### Clinicopathological Data

According to histologic type, NSCLC cases were classified as follows: (1) squamous cell carcinoma (SQCC) (histologic codes 8052, 8070-8075, 8083, 8084, 8123); (2) adenocarcinoma (AD) (histologic codes 8244, 8245, 8250-8255, 8260, 8290, 8310, 8323, 8333, 8480, 8481, 8490, 8507, 8550, 8570, 8571, 8574, and 8576); and (3) large cell carcinoma (histologic codes 8012-8014). According to the SEER criteria, all 6,395 patients with stage IA NSCLC were classified as having undergone SBRT (using regional treatment modality-specific codes) or ablation, defined as laser ablation/cryotherapy (SEER surgical code 12) and electrocautery/fulguration (includes use of hot forceps for tumor destruction; SEER surgical code 13).

### Statistical Analyses

All data were analyzed using IBM SPSS, version 20.0 (IBM Corp, Armonk, NY, USA). Kaplan-Meier analysis was performed to compare survival curves between the ablation and SBRT groups. Propensity score methods were used to control for potential differences in the baseline characteristics of the included patients. Cox regression analysis was performed to assess the balance in baseline covariates between the two groups after adjusting for the estimated propensity scores.

## Results

### Baseline Cohort Characteristics

A total of 6395 patients with stage IA NSCLC who were treated with SBRT or ablation as primary treatment from 2004 to 2015 were identified. The number of patients who received SBRT and ablation were 6004 (93.89%) and 391(6.11%), respectively. Table 1 shows the baseline characteristics of all patients, identified through the SEER database. Kaplan-Meier analyses demonstrated significant differences in overall survival (OS) between the two groups according to sex (*p*<0.001), age (p<0.001), tumor size (*p*<0.001), histologic type (*p*<0.001), differentiation grade (p<0.001), insurance status (*p*<0.001), year of diagnosis (*p*<0.001), and geographic region (*p*=0.002). However, no significant differences in OS were observed with respect to race (*p*=0.080), tumor location (*p*=0.062), laterality (*p*=0.734), marital status (p=0.340), education level (*p*=0.425), and median household income (*p*=0.531) (Table 1).

### Comparison of Disease-specific Mortality and Median Survival between the SBRT and Ablation Groups

The overall lung cancer-specific mortality rate in patients with stage IA NSCLC was 29.5% (1886/6395). The mortality rates were 29.0% (1739/6004) and 37.6% (147/391) in the SBRT and ablation groups, respectively. The overall median survival of patients with stage IA NSCLC was 20 months. The median survival in the SBRT and ablation groups were 20 and 31 months, respectively (Table 2). Compared to the SBRT group, the crude hazard ratio (HR) (95% confidence interval [CI]) was 0.931 (0.821-1.055, *p*=0.260) for patients with stage IA NSCLC in the ablation group. In patients with stage IA SQCC, the HR (95% CI) was 0.877 (0.684-1.124, *p*=0.299) in the ablation group, compared to the SBRT group. In patients with stage IA AD, the HR (95% CI) in the ablation group was 0.919 (0.768-1.099, *p*=0.353), compared to the SBRT group (Table 3).

### Kaplan-Meier Analysis of Survival Curves between the SBRT and Ablation Groups

**Figure 1:**
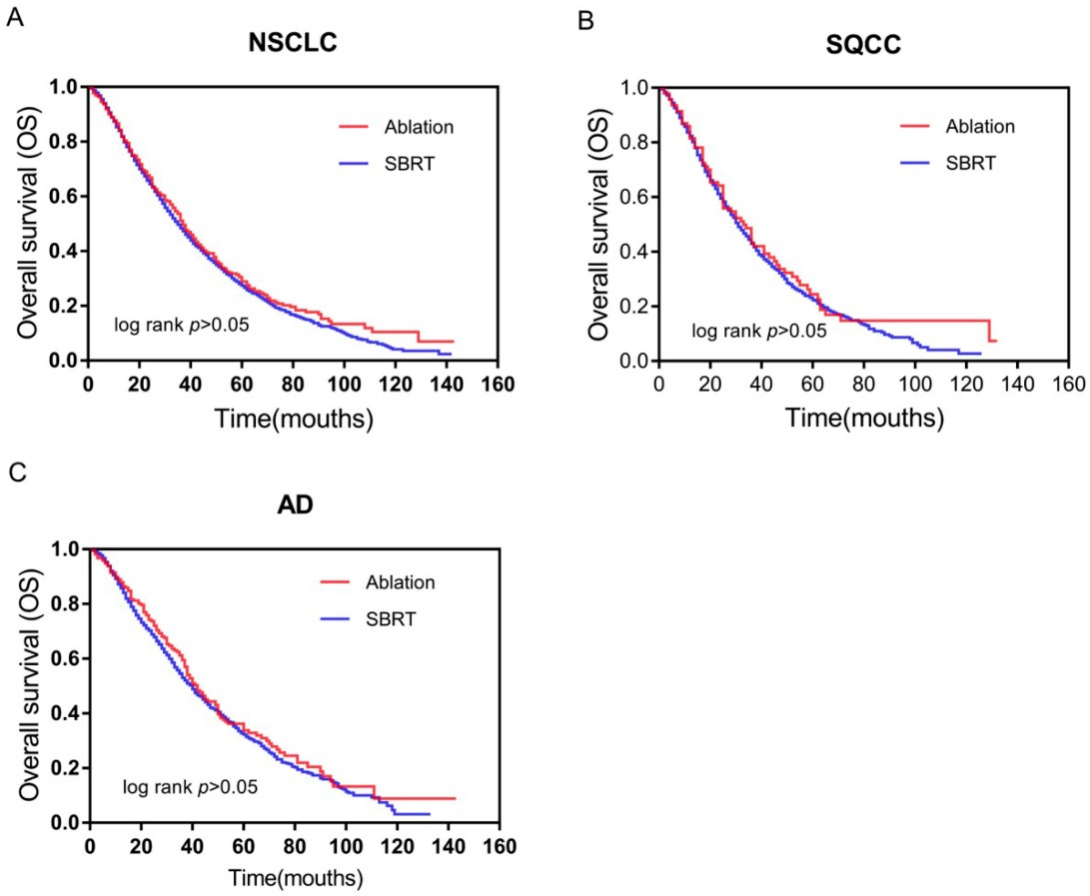
Survival curves based on Kaplan-Meier analysis for comparing between SBRT and ablation. (A) OS (p>0.05) of patients with early-stage NSCLC; (B) OS (p>0.05) of patients with early-stage SQCC; and (C) OS (p>0.05) of patients with early-stage AD. **Abbreviations**: OS, overall survival; NSCLC, non-small cell lung cancer; SBRT, stereotactic body radiotherapy; SQCC, squamous cell carcinoma; AD, adenocarcinoma.

No significant differences in survival curves were observed between the SBRT and ablation groups on Kaplan-Meier analysis, as shown in Figure 1. Among patients with stage IA NSCLC, survival (log-rank *p*>0.05) was similar in the ablation and SBRT groups (Figure 1A). Consistently, no significant differences in survival were observed between the two subtypes of NSCLC: SQCC (log-rank *p*>0.05) (Figure 1B) and AD (log-rank *p*>0.05) (Figure 1C). Our data demonstrated similar effects on survival of patients with stage IA SQCC and AD in the SBRT and ablation groups.

### Comparison of the Effects on Survival of Patients with Stage IA NSCLC between the SBRT and Ablation Groups

No significant differences (*p*=0.260) in the OS of patients with stage IA NSCLC were observed between the SBRT and ablation groups on univariate analysis (Table 3). Furthermore, no significant differences were observed in the OS of patients with stage IA SQCC (*p*=0.299) and AD (*p*=0.353) between the two groups (Table 3). A Cox model with nine variables, including sex, age, differentiation grade, tumor size, histologic type, insurance status, year of diagnosis, geographic region, and treatment, showed an HR (95% CI) of 0.930 (0.817-1.058, *p*=0.269) on comparing between the ablation and SBRT groups (Table 4). Then the following variables were excluded: insurance status, year of diagnosis, and geographic region (these covariates were not very close to the clinic), and a new Cox model was adjusted for age, sex, tumor size, differentiation grade, histologic type, and treatment.

In this model, the HR (95% CI) was 0.974 (0.858-1.105, p=0.680) on comparing between the ablation and SBRT groups (Table 5). These results indicated that no significant difference was observed between the effect of SBRT and ablation on the OS of patients with stage IA NSCLC.

## Discussion

As some elderly patients with cardiopulmonary insufficiency or other comorbidities are not eligible for surgical treatment, non-invasive options, such as SBRT and ablation, have played an increasingly important role in the treatment of NSCLC^12^,^13^. Currently, no large-scale clinical trials have compared the therapeutic effect between ablation and SBRT and no independent cohorts which can be downloaded from publicly available databases to validate our main findings and conclusions, primarily owing to the novelty and limitations associated with the practical application of ablation treatment. Recently, Johannes Uhlig^11^ conducted a retrospective study and reported that the estimated 1-, 3-, and 5-year OS rates of patients treated with ablation were comparable to those of patients treated with SBRT (1-year, 85.4% vs. 86.3%,* p*=0.76; 3-year, 47.8% vs. 45.9%, *p*=0.32; 5-year, 24.6% vs. 26.1%, *p*=0.81). These results were similar to our findings. According to our study, no significant survival difference was seen on analyzing a large SEER dataset, which contained the information of patients diagnosed with stage IA NSCLC during 2005-2014. Our results provide a curative reference on the non-inferior survival benefits achieved with ablation as primary treatment for stage IA NSCLC.

At present, it is widely accepted that SBRT is the optimal curative approach for medically inoperable patients^14^. SBRT can reach occult regional and deep structures that are difficult to explore via surgery, resulting in prolonged survival. However, SBRT has a drawback; SBRT is associated with poor control of local pulmonary lesions or multiple metastatic disease, increasing the risk of cancer-specific death. Pneumonitis, dyspnea, and chest pain were most commonly reported adverse events associated with SBRT^15^, usually occurring approximately 4-12 weeks after treatment, in a systematic review^16^,^17^. The incidence of toxicities induced by SBRT was higher in patients with central lung cancer (close to the airway) than in those with peripheral lung cancer^18^,^19^. In addition, patients with advanced age or multiple comorbidities tend to forego definitive treatment; this is one of the predominant reasons why the systemic adverse reactions of SBRT commonly appear gradually over long-term treatment. and would not become new detective reflection factors within 30 days of readmission to hospital^18^. Relatively, complications of ablation usually occur on the same day or within a few days of treatment; of these adverse events, self-limiting pneumothorax is the most common^10^,^20^. The majority of patients can experience relief after symptomatic treatment, and only a small proportion of patients (10%-30%) need to undergo chest tube placement^20^,^21^. Other rare complications of ablation include pulmonary hemorrhage^22^,^23^, air embolism^24^,^25^, pleural effusion^26^, bronchopleural fistula formation^27^, bronchospasm^10^, and sometimes even death^7^. Considering the similarity in survival rates, fewer complications and better quality of life may be the main factors influencing the choice between SBRT and ablation for inoperable patients with stage IA NSCLC. This will be a direction for future research.

Although the SEER database provides a significant data-collecting platform for addressing this urgent issue, this investigation has some limitations. Although we conducted accurate matching of cohorts, this study was retrospective in nature; thus, the factors not included in the matching process may be responsible for the observed differences in outcome. In addition, OS was analyzed without any adjustment for radiation dose, toxicities, pulmonary function, cause of death, and local progression-free survival. Furthermore, the SEER database does not provide details regarding repetitive ablation treatments and feasible approaches, such as surgical, percutaneous, and bronchoscopic ablation. Therefore, further research is needed on this topic.

Due to its retrospective design, our study has some limitations. For example, the lack of original datum from our own studies as well as validation for main findings and conclusion. Nevertheless, with the inclusion of 15 variables and nearly 6400 patients in our cohort, the present study represents a well-balanced analysis of Ablation or SBRT treatment methods. Thus, in the absence of data from prospective trials, our findings can provide information that is useful for the management in inoperable patients with stage IA NSCLC.

## Conclusion

According to the results of our study, no significant difference was observed in survival between inoperable patients with stage IA NSCLC who were treated with SBRT and ablation. Therefore, the quality of life after SBRT or ablation may be the main consideration for choosing the treatment method.

## Figures and Tables

**Table 1:** Baseline Characteristics of Patients with Stage IA NSCLC Treated with SBRT and Ablation in the SEER Program, 2004-2015

Characteristics	NSCLC		SBRT		Ablation		* p*
	Number	%	Number	%	Number	%	
Age, year							<0.001
<45	6	0.1	6	0.1	0	0	
≥45, <55	119	1.9	110	1.8	9	2.3	
≥55, <65	785	12.3	730	12.2	55	14.1	
≥65, <75	2090	32.7	1964	32.7	126	32.2	
≥75	3395	53.0	3194	53.2	201	51.4	<0.001
Sex							
Female	3607	56.4	3387	56.4	220	56.3	
Male	2788	43.6	2617	43.6	171	43.7	0.080
Race							
White	5331	83.4	5156	85.9	175	44.7	
Black	776	12.1	574	9.6	202	51.7	
Others	279	4.4	265	4.4	14	3.6	
Unknown	9	0.1	9	0.1	0	0	<0.001
Tumor size, cm							
≤1	325	5.1	272	4.5	53	13.6	
>1, ≤2	3135	49.0	2926	48.7	209	53.5	
>2, ≤3	2917	45.6	2795	46.6	122	31.2	
Unknown	18	0.3	11	0.2	7	1.7	0.062
Tumor location							
Upper lobe	3945	61.7	3715	61.9	230	58.8	
Middle lobe	293	4.6	270	4.5	23	5.9	
Lower lobe	2023	31.6	1892	31.5	131	33.5	
NOS	101	1.6	96	1.6	5	1.3	
Overlapping lesion	11	0.2	11	0.2	0	0	
Main bronchus	22	0.3	20	0.3	2	0.5	<0.001
Differentiated grade							
Grade I	548	8.6	503	8.4	45	11.5	
Grade II	1054	16.5	994	16.6	60	15.3	
Grade III	1283	20.1	1212	20.2	71	18.2	
Grade IV	35	0.5	33	0.5	2	0.5	
Unknown	3475	54.3	3262	54.3	213	54.5	<0.001
Histologic type							
Squamous cell carcinoma	2112	33.0	2017	33.6	95	24.3	
Adenocarcinoma	3113	48.7	2897	48.3	216	55.2	
Large cell carcinoma	71	1.1	68	1.1	3	0.8	
Other	1099	17.2	1022	17.0	77	19.7	0.734
Laterality							
Right-origin of primary	3647	57.0	3418	56.9	229	58.6	
Left-origin of primary	2748	43.0	2586	43.1	162	41.4	<0.001
Insurance status							
Medicaid	650	10.2	627	10.4	23	5.9	
Uninsured	32	0.5	30	0.5	2	0.5	
Unknown	813	12.7	705	11.7	108	27.6	
Insured	4900	76.6	4642	77.4	258	66.0	0.340
Marital status							
Married	2753	43.0	2582	43.0	171	43.7	
Single	695	10.9	650	10.9	45	11.5	
Divorced	841	13.2	786	13.1	55	14.1	
Widowed	1817	28.4	1707	28.4	110	28.1	
Unknown	280	4.4	270	4.5	10	2.6	
Unmarried or Domestic Partner	9	0.1	9	0.1	0	0	<0.001
Year of diagnosis							
2004-2007	1013	15.8	866	14.4	147	37.6	
2008-2011	1969	30.8	1814	30.2	155	39.6	
2012-2015	3413	53.4	3324	55.4	89	22.8	0.002
Geographic region							
East	2799	43.8	2673	44.6	126	32.2	
Northwest	11	0.2	11	0.2	0	0	
West	2532	39.6	2301	38.3	231	59.1	
North	882	13.7	855	14.2	27	6.9	
Southwest	171	2.7	164	2.7	7	1.8	0.425
High school education							
≥21	841	13.2	758	12.6	83	21.2	
13-20	1933	30.2	1810	30.1	123	31.5	
7-12	3103	48.5	2952	49.2	151	38.6	
<7	518	8.1	484	8.1	34	8.7	
Median household income (dollar, in tons)							0.531
<38000	361	5.6	356	5.9	5	1.3	
38000-47999	1031	16.2	1019	17.0	12	3.1	
48000-62999	2433	38.1	2245	37.4	188	48.1	
>63000	2570	40.1	2384	39.7	186	47.6	
Total	6395	100.0	6004	100.0	391	100.0	

**Abbreviations:** NSCLC, non-small cell lung cancer; SBRT, stereotactic body radiotherapy; NOS, not otherwise specified; SEER: surveillance, epidemiology and end Result.

**Table 2:** Association with Cancer-Specific Mortality and Median Survival Time Among Patient Groups (SEER database, 2004-2015)

Group	Mortality, n/N (%)	Median survival time (months)
Overall	29.5% (1886/6395)	20
SBRT	29.0% (1739/6004)	20
Ablation	37.6% (147/391)	31

**Abbreviations:** SEER, Surveillance, Epidemiology and End Results; SBRT, stereotactic body radiotherapy.

**Table 3:** Univariate Analysis Comparing Patient Survival (*SBRT vs* Ablation)

Variable	Number	Univariable Analysis		
		HR	95%CI	*p*
NSCLC	6395	0.931	0.821-1.055	0.260
SQCC	2112	0.877	0.684-1.124	0.299
AD	3113	0.919	0.768-1.099	0.353

**Abbreviations:** SBRT, stereotactic body radiotherapy; NSCLC, non-small cell lung cancer; SQCC, Squamous cell carcinoma; AD, Adenocarcinoma; HR, hazard ratio; CI, confidence interval.

**Table 4:** Multivariate Analysis Using a Cox Proportional Hazards Model in Patients with stage IA NSCLC

Variable	Multivariable Analysis		
	HR	95% CI	*p*
Age, year			<0.001
<45	Reference		
≥45, <55	4.020	0.977 -16.539	0.054
≥55, <65	4.091	1.016 -16.466	0.047
≥65, <75	4.872	1.213 -19.566	0.026
≥75	5.244	1.306 -21.054	0.019
Sex			<0.001
Female	Reference		
Male	1.288	1.201 -1.380	
Tumor size, cm			<0.001
≤1	Reference		
>1, ≤2	1.110	0.938 -1.313	0.224
>2, ≤3	1.242	1.049 -1.471	0.012
Unknown	2.655	1.602 -4.399	<0.001
Differentiated grade			0.003
Grade I	Reference		
Grade II	1.306	1.114 -1.532	0.001
Grade III	1.320	1.129 -1.542	<0.001
Grade IV	1.211	0.794 -1.847	0.374
Unknown	1.187	1.028 -1.370	0.019
Histologic type			<0.001
Squamous cell carcinoma	Reference		
Adenocarcinoma	0.821	0.757 -0.891	<0.001
Large cell carcinoma	1.190	0.883 -1.605	0.253
Other	0.923	0.836 -1.018	0.110
Insurance status			0.001
Medicaid	Reference		
Uninsured	0.893	0.501 -1.592	0.701
Unknown	0.871	0.735 -1.031	0.109
Insured	0.782	0.694 -0.882	<0.001
Year of diagnosis			<0.001
2004-2007	Reference		
2008-2011	0.818	0.722 -0.926	0.002
2012-2015	0.738	0.644 -0.845	<0.001
Geographic region			0.005
East	Reference		
Northwest	2.282	1.081 -4.817	0.030
West	0.896	0.830 -0.967	0.005
North	0.895	0.805 -0.995	0.040
Southwest	0.901	0.724 -1.122	0.352
Treatment			0.269
SBRT	Reference		
Ablation	0.930	0.817 -1.058	

**Notes:** ^a^ Multivariate analysis for age, sex, tumor size, tumor location, differentiated grade, histologic type, insurance status, year of diagnosis, geographic region and treatment. **Abbreviations:** NSCLC, non-small cell lung cancer; NOS, not otherwise specified; HR, hazard ratio; CI, confidence interval; SBRT, stereotactic body radiotherapy.

**Table 5:** Univariable and Multivariable Analyses on OS in Patients with stage IA NSCLC

Variable	Univariable analysis			Multivariable analysis ^a^		
	HR	95% CI	*p*	HR	95% CI	*p*
Age, year			<0.001			0.001
<45	Reference			Reference		
≥45, <55	4.020	0.977 -16.539	0.054	3.443	0.837 -14.158	0.087
≥55, <65	4.091	1.016 -16.466	0.047	3.364	0.836 -13.533	0.088
≥65, <75	4.872	1.213 -19.566	0.026	3.886	0.968 -15.593	0.056
≥75	5.244	1.306 -21.054	0.019	4.169	1.039 -16.720	0.044
Sex			<0.001			<0.001
Female	Reference			Reference		
Male	1.288	1.201 -1.380		1.280	1.195 -1.372	
Tumor size, cm			<0.001			<0.001
≤1	Reference			Reference		
>1, ≤2	1.110	0.938 -1.313	0.224	1.126	0.952 -1.332	0.165
>2, ≤3	1.242	1.049 -1.471	0.012	1.283	1.084 -1.519	0.004
Unknown	2.655	1.602 -4.399	<0.001	3.035	1.835 -5.018	<0.001
Differentiated grade			0.003			0.004
Grade I	Reference			Reference		
Grade II	1.306	1.114 -1.532	0.001	1.307	1.115 -1.533	0.001
Grade III	1.320	1.129 -1.542	<0.001	1.324	1.133 -1.546	<0.001
Grade IV	1.211	0.794 -1.847	0.374	1.311	0.860 -1.997	0.208
Unknown	1.187	1.028 -1.370	0.019	1.202	1.042 -1.387	0.012
Histologic type			<0.001			<0.001
Squamous cell carcinoma	Reference			Reference		
Adenocarcinoma	0.821	0.757 -0.891	<0.001	0.810	0.747 -0.879	<0.001
Large cell carcinoma	1.190	0.883 -1.605	0.253	1.256	0.931 -1.694	0.135
Other	0.923	0.836 -1.018	0.110	0.958	0.869 -1.056	0.384
Treatment			0.269			0.680
SBRT	Reference			Reference		
Ablation	0.930	0.817 -1.058		0.974	0.858 -1.105	

**Notes:** ^a^ Multivariate analysis for age, sex, tumor size, differentiated grade, histologic type, treatment. **Abbreviations:** OS, overall survival; NSCLC, non-small cell lung cancer; HR, hazard ratio; CI, confidence interval; SBRT, stereotactic body radiotherapy.
